# Notes and News

**Published:** 1896-07-11

**Authors:** 


### Notes and News.

(Collected and Communicated.)

### HOSPITAL MEETINGS.

Small-Pox and Vaccination Hospital, Highgate Hill.—The half-yearly General Court of Governors will be held at the Hospital on Tuesday, July 14th, at 5 p.m.

We are glad to learn that during the last three days no fresh cases of small-pox have occurred at Gloucester.

The military charities will benefit to the extent of about £3,500 as a result of the military tournament at Islington, which proved more popular than ever this year.

A testimonial is to be presented to Sir William Broadbent, upon his retiring from the post of senior physician at St. Mary's Hospital, in recognition of his valuable services to the institution. Mr. Malcolm Morris, 8, Harley Street, will receive contributions, which are restricted to two guineas.

Very strong measures have been taken at Flushing, in the United States, to stamp out a severe epidemic of measles. The health officers quarantined all premises where the disease was known to exist, and a large signboard with the word "Measles" painted upon it was hung over the door.

A prize for industrial hygiene is offered by the council of the Society of Arts under the terms of the Benjamin Shaw Trust, to consist of a gold medal or a donation of £20. The prize will be awarded "for any discovery, invention, or newly-devised method of obviating or materially diminishing any risk to life, limb, or health, incidental to any industrial occupation, and not previously capable of being obviated or diminished by any known and practically available means." Intending competitors should send in descriptions of their inventions not later than December 31st, 1896, to the secretary of the Society of Arts, Adelphi, London, W.C.

The British Medical Association meets this year at Carlisle during the last week in July. Next year it meets at Montreal.

The Prince of Wales has consented to open the convalescent home at Limpsfield, which is in connection with the Charing Cross Hospital, on Saturday next.

H.R.H. the Prince of Wales, accompanied by the Princess of Wales, will open the Charing Cross Hospital Convalescent Home at Limpsfield, on Saturday next, at twelve o'clock.

A man is now in the service of the Italian Government who has undergone Maragliano's treatment for consumption. It is claimed that he was suffering from advanced tuberculosis, and that after some months' treatment, recovery has taken place.

Some of our contemporaries have announced the fact that a certain number of millionaires have subscribed £500,000 to Guy's Hospital Endowment Fund. The original paragraph was meant as a joke; but, being misinterpreted, the authorities have received congratulations instead of contributions from several sources. The actual amount subscribed is £169,000.

The Hospital Saturday Fund collection at Sydney, New South Wales, was carried on with great vigour this year. Unfortunately the objectionable street begging was resorted to, many of the ladies of Sydney taking an active part in the day's proceedings. However much we may disapprove of the method of the collection, we are glad to say that the hospitals will benefit to the extent of nearly £4,000.

At a meeting of the East India Association this week the medical needs of India were under discussion. Dr. Bahadurji stated that the system of civil medical administration did not work successfully in meeting the growing requirements of the population. Lord Reay said that until the medical schools were placed on the same footing as medical schools in other parts of the world, an injustice was done to the medical students in India, which should be removed. He thought the Civil Medical Service should be entirely independent of the Army Medical Service.

During the last few weeks a large number of bazaars have been held in London for charitable objects, and some of them with success wholly inadequate to the trouble expended upon them. For some time past those occupied in promoting entertainments to secure funds for institutions have realized that the bazaar pure and simple no longer offers any attraction to a London public, especially during a gay and hot season, and, consequently, the most astonishing amount of ingenuity is exercised to secure entertainments and displays which will tempt the reluctant public firstly to lend their presence and then to part with their money. In some cases a goodly sum has resulted from the entertainment, but at the best one entirely out of all proportion to the amount of energy and trouble expended by the promoters, and quite ridiculous in contrast to that produced by a country function of the same kind. In the country local interest can be made to centre round a bazaar so effectively that really substantial additions are added to the funds of needy charities. In London, however, we feel that the ingenuity and trouble expended can be much better utilised than in multiplying unsatisfactory bazaars. Friends of many institutions realise this, but there is still scope for those who possess an inventive faculty to introduce entertainments which will attract the public who can spend.

